# Supplementary material for: A Likelihood Approach for Real-Time Calibration of Stochastic Compartmental Epidemic Models
Source: PLoS Comput Biol. 2017 Jan 17;13(1):e1005257. doi: 10.1371/journal.pcbi.1005257 (PMC5240920; doi:10.1371/journal.pcbi.1005257)
Supplement: S1 File — (TAR.GZ) [file pcbi.1005257.s014.tar.gz › HSPH_Online-SI-Revision/Additions/EnKF08/n100-extreme_noisy/n100-extreme_noisy_EnKF2_table-8.pdf]

1	newly 3 weeks spec	1833.	1049.06	{272., 1675.}	66.8325	0.480199	newly 3 weeks cumul	3045.73	21 136.8	{8664., 66 916.}	596.508	0.692846	newly 5 weeks spec	5869.58	129.95	{4., 215.}	97.786	2.15483	newly 5 weeks cumul	12 428.	21 617.2	{8782., 67 239.}	102.639	0.248004	newly 62 weeks cumul	94 760.2	21 711.7	{8786., 67 243.}	77.4668	0.803784
2	newly 3 weeks spec	2838.	1394.2	{103., 5925.}	91.1755	0.8123	newly 3 weeks cumul	5925.	7544.58	{2438., 25 158.}	93.6358	0.2962	newly 5 weeks spec	5914.	677.58	{5., 1568.}	93.0264	1.76491	newly 5 weeks cumul	16 050.	9186.14	{2897., 35 212.}	84.4325	0.568805	newly 58 weeks cumul	82 551.	11 020.9	{3113., 38 060.}	86.9933	1.12348
3	newly 3 weeks spec	1557.	2511.73	{0., 7226.}	161.816	1.42914	newly 3 weeks cumul	2607.	29 610.4	{7241., 69 242.}	1039.12	0.930361	newly 5 weeks spec	6571.	1020.83	{0., 3481.}	84.4646	2.51193	newly 5 weeks cumul	12 454.	32 151.	{7511., 69 242.}	177.695	0.417908	newly 63 weeks cumul	90 095.	35 326.4	{7536., 69 242.}	63.9305	0.549132
4	newly 3 weeks spec	302.	10 728.7	{1147., 20 619.}	3455.33	1.3676	newly 3 weeks cumul	421.	46 518.7	{6785., 96 101.}	10 949.6	1.89416	newly 5 weeks spec	3492.	5621.12	{20., 12 171.}	117.351	0.696722	newly 5 weeks cumul	5026.	60 044.5	{13 668., 99 572.}	1097.65	0.983865	newly 67 weeks cumul	90 300.	74 387.5	{18 351., 106 467.}	28.9612	0.205282
5	newly 3 weeks spec	841.	6219.95	{947., 11 013.}	643.537	0.730376	newly 3 weeks cumul	1316.	39 016.9	{13 422., 89 467.}	2865.03	1.35578	newly 5 weeks spec	3644.	1937.83	{76., 3661.}	63.037	0.661887	newly 5 weeks cumul	6776.	44 328.6	{14 208., 104 584.}	556.671	0.721801	newly 64 weeks cumul	88 461.	47 480.	{14 585., 107 032.}	56.2143	0.416684
6	newly 3 weeks spec	1468.	1079.6	{280., 1927.}	61.846	0.408932	newly 3 weeks cumul	2440.	22 954.1	{6719., 68 642.}	841.576	0.795148	newly 5 weeks spec	5118.	253.14	{4., 233.}	95.0539	2.09861	newly 5 weeks cumul	10 351.	23 619.6	{8996., 69 046.}	138.208	0.255244	newly 62 weeks cumul	91 404.	24 497.6	{9145., 69 063.}	75.1422	0.739644
7	newly 3 weeks spec	1624.	2664.92	{2., 8969.}	217.183	1.30961	newly 3 weeks cumul	2704.	8616.82	{856., 47 386.}	282.683	0.402493	newly 5 weeks spec	5875.	882.32	{0., 1912.}	93.8485	2.5413	newly 5 weeks cumul	11 979.	11 315.9	{860., 55 800.}	137.519	0.826596	newly 60 weeks cumul	90 884.	11 976.1	{860., 63 680.}	87.3311	1.49058
8	newly 3 weeks spec	1359.	1268.55	{262., 1699.}	88.4393	0.460503	newly 3 weeks cumul	2461.	22 060.7	{8498., 50 762.}	797.024	0.797244	newly 5 weeks spec	5170.	162.18	{3., 220.}	96.8631	2.21259	newly 5 weeks cumul	10 407.	22 652.4	{8613., 55 645.}	131.171	0.256251	newly 62 weeks cumul	92 361.	22 789.7	{8613., 56 455.}	75.7085	0.768149
9	newly 3 weeks spec	953.	1356.54	{280., 4061.}	112.842	0.407695	newly 3 weeks cumul	1486.	20 465.7	{5618., 66 301.}	1281.71	0.967765	newly 5 weeks spec	4055.	275.95	{6., 356.}	94.2163	1.98965	newly 5 weeks cumul	7477.	21 262.5	{6624., 68 512.}	194.382	0.352262	newly 62 weeks cumul	92 411.	21 749.6	{7224., 68 624.}	76.4827	0.833363
10	newly 3 weeks spec	629.	11 201.3	{1457., 21 546.}	1690.36	1.13978	newly 3 weeks cumul	952.	54 268.2	{13 484., 94 748.}	5603.67	1.64942	newly 5 weeks spec	4393.	4846.32	{42., 10 381.}	73.8056	0.609757	newly 5 weeks cumul	6990.	66 523.6	{22 045., 109 809.}	856.675	0.921161	newly 66 weeks cumul	75 317.	77 164.6	{26 737., 111 827.}	44.6792	0.245053
11	newly 3 weeks spec	2514.	785.96	{7., 554.}	100.485	1.32672	newly 3 weeks cumul	4845.	5601.57	{1090., 9943.}	121.093	0.489321	newly 5 weeks spec	6044.	259.53	{0., 217.}	97.0299	2.49677	newly 5 weeks cumul	15 023.	6332.43	{1090., 10 009.}	95.1701	0.846332	newly 60 weeks cumul	84 746.	6616.53	{1090., 10 013.}	92.7998	1.50299
12	newly 3 weeks spec	1011.	618.31	{0., 817.}	108.958	1.34641	newly 3 weeks cumul	1634.	8084.1	{1297., 20 396.}	408.394	0.551402	newly 5 weeks spec	4508.	282.92	{0., 262.}	99.2311	2.87422	newly 5 weeks cumul	8400.	8793.78	{1337., 20 417.}	79.9555	0.390755	newly 61 weeks cumul	92 398.	9194.12	{1340., 20 417.}	90.0494	1.24823
13	newly 3 weeks spec	2062.	1741.79	{110., 4351.}	130.549	0.912224	newly 3 weeks cumul	3828.	8307.96	{546., 14 483.}	194.877	0.435695	newly 5 weeks spec	4926.	587.26	{14., 1983.}	90.2716	1.7572	newly 5 weeks cumul	12 079.	9854.	{668., 21 304.}	118.975	0.760033	newly 59 weeks cumul	90 371.	12 035.9	{791., 59 134.}	88.3309	1.43592
14	newly 3 weeks spec	207.	8022.55	{837., 20 741.}	3778.44	1.36529	newly 3 weeks cumul	282.	46 982.6	{12 758., 95 255.}	16 560.7	2.06014	newly 5 weeks spec	1855.	2489.58	{53., 4098.}	110.381	0.641501	newly 5 weeks cumul	2833.	53 743.6	{13 284., 106 723.}	1800.36	1.15428	newly 64 weeks cumul	71 218.	57 280.5	{13 363., 110 849.}	55.514	0.360829
15	newly 3 weeks spec	874.	5977.78	{972., 14 867.}	588.105	0.686137	newly 3 weeks cumul	1434.	43 686.8	{11 185., 98 006.}	2948.38	1.33877	newly 5 weeks spec	4922.	1497.64	{81., 3578.}	71.4072	0.864925	newly 5 weeks cumul	8480.	48 054.5	{11 930., 105 184.}	471.912	0.651049	newly 64 weeks cumul	79 509.	50 457.1	{13 993., 107 274.}	53.6608	0.388132
16	newly 3 weeks spec	582.	7724.71	{952., 20 565.}	1227.27	0.93978	newly 3 weeks cumul	805.	48 171.3	{13 879., 96 000.}	5884.01	1.65637	newly 5 weeks spec	4823.	2308.45	{72., 4219.}	73.8103	0.780931	newly 5 weeks cumul	7337.	54 597.7	{15 515., 107 478.}	645.484	0.763363	newly 64 weeks cumul	91 266.	58 102.6	{15 907., 111 416.}	48.9349	0.357396
17	newly 3 weeks spec	2320.	1329.14	{129., 2863.}	109.676	0.897583	newly 3 weeks cumul	4411.	8116.55	{1379., 10 970.}	171.125	0.416151	newly 5 weeks spec	5419.	306.4	{9., 1591.}	94.3458	1.85459	newly 5 weeks cumul	13 371.	9036.37	{1555., 13 693.}	109.106	0.734585	newly 59 weeks cumul	87 876.	9821.34	{1603., 18 142.}	89.219	1.39655
18	newly 3 weeks spec	1347.	1796.18	{3., 1617.}	187.433	1.2253	newly 3 weeks cumul	2379.	7215.42	{1196., 15 880.}	264.266	0.364973	newly 5 weeks spec	4153.	520.63	{0., 251.}	98.046	2.57737	newly 5 weeks cumul	9012.	8804.69	{1202., 18 862.}	135.077	0.728791	newly 60 weeks cumul	79 943.	9209.11	{1202., 19 316.}	88.845	1.48971
19	newly 3 weeks spec	927.	1641.85	{284., 3377.}	130.666	0.387094	newly 3 weeks cumul	1494.	24 053.3	{8328., 59 397.}	1513.35	1.04367	newly 5 weeks spec	4254.	315.21	{5., 671.}	92.5969	1.89639	newly 5 weeks cumul	7918.	25 056.8	{8441., 60 857.}	223.779	0.386622	newly 62 weeks cumul	93 229.	25 346.7	{8447., 61 381.}	75.5704	0.772283
20	newly 3 weeks spec	53.	10 582.2	{843., 20 831.}	19 866.4	2.09236	newly 3 weeks cumul	74.	50 465.4	{11 244., 98 988.}	68 096.5	2.72166	newly 5 weeks spec	1059.	5431.44	{6., 12 195.}	454.755	0.969956	newly 5 weeks cumul	1395.	63 611.4	{13 820., 102 234.}	4459.96	1.56569	newly 67 weeks cumul	91 568.	75 878.9	{16 674., 106 627.}	27.457	0.203673
21	newly 3 weeks spec	845.	6669.36	{1091., 14 312.}	692.166	0.782141	newly 3 weeks cumul	1345.	48 939.2	{13 435., 121 027.}	3540.16	1.44857	newly 5 weeks spec	4282.	1770.88	{100., 3961.}	71.4629	0.747353	newly 5 weeks cumul	7677.	53 970.1	{15 036., 124 232.}	604.907	0.757811	newly 64 weeks cumul	83 732.	56 850.1	{15 793., 124 459.}	49.922	0.34103
22	newly 3 weeks spec	804.	5939.2	{742., 11 038.}	649.256	0.750866	newly 3 weeks cumul	1237.	53 325.6	{14 792., 107 759.}	4212.31	1.53403	newly 5 weeks spec	4322.	1612.11	{7., 4039.}	70.8165	1.07989	newly 5 weeks cumul	7480.	57 955.6	{22 644., 113 891.}	678.755	0.826319	newly 65 weeks cumul	84 795.	60 503.4	{27 595., 114 525.}	43.449	0.298231
23	newly 3 weeks spec	2606.	949.22	{65., 1032.}	84.0952	0.804446	newly 3 weeks cumul	5006.	7200.66	{2559., 5163.}	103.61	0.254946	newly 5 weeks spec	5905.	348.31	{1., 596.}	94.1014	1.87119	newly 5 weeks cumul	15 055.	8105.94	{2856., 6082.}	87.7067	0.584225	newly 58 weeks cumul	84 036.	8997.36	{2971., 6871.}	89.3129	1.19126
24	newly 3 weeks spec	1803.	1105.88	{25., 2053.}	84.7621	0.674223	newly 3 weeks cumul	3172.	6840.19	{2707., 11 479.}	122.889	0.191266	newly 5 weeks spec	5220.	393.46	{1., 1043.}	92.4625	1.76642	newly 5 weeks cumul	11 528.	7872.86	{3196., 15 058.}	77.7094	0.439216	newly 58 weeks cumul	92 404.	8718.36	{3228., 19 110.}	90.565	1.19107
25	newly 3 weeks spec	784.	658.36	{0., 574.}	128.01	1.3867	newly 3 weeks cumul	1188.	7140.2	{701., 12 110.}	516.466	0.628379	newly 5 weeks spec	4655.	218.45	{0., 201.}	97.0082	2.94305	newly 5 weeks cumul	7743.	7745.27	{776., 13 268.}	77.482	0.428435	newly 61 weeks cumul	89 551.	8051.48	{790., 15 463.}	91.0091	1.32832
26	newly 3 weeks spec	2337.	1724.07	{158., 4217.}	121.923	0.834989	newly 3 weeks cumul	4561.	7763.01	{1215., 17 426.}	163.709	0.441082	newly 5 weeks spec	5505.	705.87	{12., 1595.}	92.3319	1.62599	newly 5 weeks cumul	13 758.	9570.33	{1527., 27 022.}	110.778	0.73421	newly 59 weeks cumul	89 914.	11 420.5	{1785., 28 480.}	89.2536	1.35471
27	newly 3 weeks spec	3178.	1884.59	{0., 1541.}	125.175	1.87942	newly 3 weeks cumul	6240.	6864.88	{527., 24 752.}	130.477	0.642465	newly 5 weeks spec	6900.	713.75	{0., 397.}	93.0596	2.70516	newly 5 weeks cumul	18 218.	8920.83	{527., 25 183.}	101.211	0.96056	newly 60 weeks cumul	90 939.	10 083.1	{527., 34 065.}	88.9123	1.51685
28	newly 3 weeks spec	1642.	6427.96	{906., 12 966.}	310.117	0.530426	newly 3 weeks cumul	2833.	48 079.8	{12 139., 119 483.}	1598.71	1.08151	newly 5 weeks spec	6075.	1765.54	{70., 3942.}	75.5516	0.942245	newly 5 weeks cumul	12 201.	53 064.1	{13 886., 122 570.}	341.461	0.544378	newly 64 weeks cumul	93 780.	55 861.1	{15 054., 122 904.}	54.351	0.411361
29	newly 3 weeks spec	2186.	1185.56	{110., 1048.}	86.4547	0.677256	newly 3 weeks cumul	4260.	7770.75	{2795., 15 670.}	111.227	0.198003	newly 5 weeks spec	5160.	422.83	{2., 660.}	91.8859	1.67371	newly 5 weeks cumul	12 858.	8891.57	{3188., 20 360.}	85.187	0.474502	newly 58 weeks cumul	82 934.	9758.28	{3390., 25 291.}	88.4256	1.11934
30	newly 3 weeks spec	404.	10 484.9	{1000., 20 838.}	2499.55	1.24105	newly 3 weeks cumul	607.	43 991.6	{9840., 87 735.}	7147.37	1.73534	newly 5 weeks spec	3670.	5791.41	{15., 12 199.}	116.714	0.724951	newly 5 weeks cumul	5636.	57 724.8	{15 200., 96 117.}	926.438	0.928517	newly 67 weeks cumul	93 251.	72 347.4	{22 779., 97 239.}	27.8403	0.200487
31	newly 3 weeks spec	169.	7416.88	{538., 19 667.}	4300.54	1.49685	newly 3 weeks cumul	246.	44 347.5	{7297., 95 772.}	17 927.4	2.08489	newly 5 weeks spec	2181.	4438.65	{0., 12 525.}	184.083	1.26171	newly 5 weeks cumul	3075.	54 474.2	{10 622., 96 220.}	1671.52	1.12172	newly 68 weeks cumul	88 759.	69 305.4	{11 982., 99 060.}	28.9987	0.238107
32	newly 3 weeks spec	874.	5969.2	{261., 11 279.}	612.947	0.787121	newly 3 weeks cumul	1385.	55 284.7	{10 508., 109 040.}	3893.18	1.46793	newly 5 weeks spec	4137.	1760.6	{0., 4108.}	77.4972	1.32503	newly 5 weeks cumul	7503.	60 139.9	{14 007., 114 773.}	709.349	0.836441	newly 65 weeks cumul	77 539.	64 420.9	{17 884., 115 424.}	43.5335	0.290618
33	newly 3 weeks spec	1347.	2400.86	{0., 8321.}	193.719	1.87961	newly 3 weeks cumul	2306.	39 653.8	{6985., 116 693.}	1625.17	1.08218	newly 5 weeks spec	4307.	695.19	{0., 2434.}	92.1911	2.85653	newly 5 weeks cumul	9125.	41 500.	{7254., 120 842.}	370.043	0.607185	newly 63 weeks cumul	92 452.	42 796.9	{7288., 121 043.}	63.072	0.57884
34	newly 3 weeks spec	2211.	771.94	{148., 744.}	85.5305	0.809206	newly 3 weeks cumul	4652.	5820.74	{2328., 5962.}	81.758	0.229249	newly 5 weeks spec	4361.	259.19	{3., 395.}	94.2786	1.858	newly 5 weeks cumul	12 123.	6503.38	{2681., 6254.}	82.2671	0.528464	newly 58 weeks cumul	74 850.	7214.09	{2830., 6291.}	90.5207	1.20695
35	newly 3 weeks spec	845.	2858.77	{0., 8339.}	336.025	1.47771	newly 3 weeks cumul	1377.	36 097.	{7121., 81 395.}	2526.05	1.27367	newly 5 weeks spec	3852.	1148.31	{0., 3585.}	89.7079	2.53654	newly 5 weeks cumul	7034.	38 973.4	{7348., 81 395.}	461.795	0.654374	newly 63 weeks cumul	93 735.	41 652.7	{7375., 99 039.}	62.0197	0.565416
36	newly 3 weeks spec	2415.	463.9	{0., 510.}	96.2791	1.69045	newly 3 weeks cumul	4659.	6248.17	{1034., 9125.}	87.6229	0.316344	newly 5 weeks spec	5307.	99.2	{0., 84.}	98.1308	2.86801	newly 5 weeks cumul	13 623.	6558.08	{1124., 9125.}	76.3653	0.596546	newly 61 weeks cumul	83 417.	6656.38	{1127., 9125.}	92.0204	1.32246
37	newly 3 weeks spec	569.	10 220.9	{2452., 21 293.}	1702.62	1.1617	newly 3 weeks cumul	794.	46 132.	{18 259., 84 539.}	5711.46	1.66466	newly 5 weeks spec	3995.	4704.37	{1037., 9834.}	74.1204	0.458098	newly 5 weeks cumul	6432.	57 767.8	{23 507., 102 488.}	800.299	0.890661	newly 66 weeks cumul	88 408.	70 251.3	{30 998., 111 365.}	37.3905	0.217313
38	newly 3 weeks spec	174.	11 337.	{2764., 21 292.}	6416.17	1.67612	newly 3 weeks cumul	245.	51 287.9	{19 356., 85 182.}	20 833.8	2.23992	newly 5 weeks spec	2064.	5213.09	{550., 9568.}	180.922	0.650107	newly 5 weeks cumul	2893.	64 264.7	{23 474., 104 871.}	2121.39	1.28658	newly 66 weeks cumul	81 546.	75 883.8	{31 891., 111 089.}	33.6321	0.174773
39	newly 3 weeks spec	1689.	1784.72	{0., 1691.}	157.936	1.50057	newly 3 weeks cumul	3181.	7504.98	{950., 20 326.}	222.456	0.50673	newly 5 weeks spec	4543.	689.58	{0., 235.}	98.6705	2.60839	newly 5 weeks cumul	10 645.	9421.53	{950., 21 236.}	126.953	0.8246	newly 60 weeks cumul	70 294.	10 262.4	{970., 21 242.}	88.2092	1.44355
40	newly 3 weeks spec	2733.	470.52	{0., 506.}	93.689	1.95614	newly 3 weeks cumul	5234.	6264.41	{797., 11 397.}	79.1935	0.355336	newly 5 weeks spec	6145.	133.3	{0., 92.}	97.8308	3.0723	newly 5 weeks cumul	15 669.	6640.86	{832., 11 677.}	73.3352	0.650956	newly 61 weeks cumul	84 270.	6871.79	{832., 11 792.}	91.8455	1.33245
41	newly 3 weeks spec	2085.	1636.15	{308., 3424.}	82.8772	0.507891	newly 3 weeks cumul	3973.	21 583.	{8602., 53 590.}	443.532	0.592936	newly 5 weeks spec	5334.	257.88	{7., 537.}	95.8208	1.97353	newly 5 weeks cumul	12 888.	22 449.2	{8732., 56 204.}	102.121	0.245713	newly 62 weeks cumul	72 211.	22 741.8	{8781., 56 801.}	69.1228	0.648993
42	newly 3 weeks spec	641.	10 008.1	{1950., 22 067.}	1468.8	1.06342	newly 3 weeks cumul	992.	50 521.7	{14 558., 92 388.}	4992.91	1.58701	newly 5 weeks spec	4341.	4358.14	{68., 10 533.}	73.759	0.657328	newly 5 weeks cumul	7191.	61 454.7	{20 094., 109 450.}	756.901	0.842725	newly 66 weeks cumul	73 182.	71 961.6	{27 171., 124 105.}	47.6644	0.243344
43	newly 3 weeks spec	1358.	1999.72	{96., 8490.}	177.202	0.769194	newly 3 weeks cumul	2359.	9466.05	{471., 18 419.}	337.738	0.373941	newly 5 weeks spec	4809.	731.68	{12., 1817.}	92.4483	1.72273	newly 5 weeks cumul	9757.	11 365.4	{580., 38 342.}	137.7	0.64438	newly 59 weeks cumul	92 457.	13 391.6	{701., 60 598.}	87.15	1.36691
44	newly 3 weeks spec	944.	5781.3	{691., 10 796.}	526.347	0.734961	newly 3 weeks cumul	1464.	50 981.5	{21 423., 103 204.}	3385.25	1.45159	newly 5 weeks spec	6185.	1530.9	{12., 3968.}	77.0416	1.15198	newly 5 weeks cumul	10 162.	55 385.5	{22 581., 103 239.}	450.514	0.705969	newly 65 weeks cumul	91 937.	57 814.1	{24 688., 103 239.}	43.5817	0.315656
45	newly 3 weeks spec	599.	5705.07	{730., 15 049.}	856.83	0.783952	newly 3 weeks cumul	910.	46 606.8	{12 323., 96 406.}	5022.1	1.5417	newly 5 weeks spec	4808.	1412.51	{36., 3579.}	74.7411	1.03279	newly 5 weeks cumul	7429.	50 733.4	{12 828., 108 499.}	588.113	0.715786	newly 64 weeks cumul	82 950.	53 517.6	{13 003., 112 721.}	52.4397	0.392962
46	newly 3 weeks spec	564.	6495.72	{1140., 20 013.}	1054.12	0.882486	newly 3 weeks cumul	898.	45 602.3	{8240., 97 465.}	4979.76	1.53209	newly 5 weeks spec	3017.	1680.46	{115., 3606.}	69.6195	0.679721	newly 5 weeks cumul	5294.	50 491.4	{9432., 109 155.}	856.532	0.838773	newly 64 weeks cumul	85 120.	52 991.9	{10 024., 113 232.}	56.4764	0.429552
47	newly 3 weeks spec	765.	9512.34	{2062., 21 014.}	1151.95	0.990145	newly 3 weeks cumul	1158.	45 642.4	{9736., 86 118.}	3842.42	1.47024	newly 5 weeks spec	3839.	4668.81	{78., 9866.}	78.4316	0.540832	newly 5 weeks cumul	6738.	56 947.8	{12 103., 101 177.}	749.9	0.865565	newly 66 weeks cumul	82 631.	70 698.2	{28 808., 111 896.}	36.7633	0.23108
48	newly 3 weeks spec	268.	10 999.4	{3163., 21 243.}	4005.31	1.50221	newly 3 weeks cumul	370.	52 706.1	{18 281., 88 057.}	14 144.9	2.07295	newly 5 weeks spec	2437.	5031.5	{606., 9740.}	134.827	0.561328	newly 5 weeks cumul	3631.	65 204.4	{24 765., 110 503.}	1695.77	1.19628	newly 66 weeks cumul	91 693.	77 559.9	{31 462., 112 006.}	31.0111	0.176454
49	newly 3 weeks spec	151.	6674.1	{287., 17 848.}	4332.78	1.39891	newly 3 weeks cumul	195.	36 595.9	{5235., 90 197.}	18 667.1	2.04811	newly 5 weeks spec	3032.	4858.55	{1., 13 737.}	153.316	1.28292	newly 5 weeks cumul	3868.	47 288.2	{6254., 91 860.}	1125.19	0.927558	newly 68 weeks cumul	88 820.	64 146.	{7704., 100 372.}	34.5802	0.303586
50	newly 3 weeks spec	2772.	1599.66	{136., 3819.}	110.074	0.940866	newly 3 weeks cumul	5085.	8440.	{1009., 15 257.}	163.172	0.47302	newly 5 weeks spec	6948.	339.46	{13., 1710.}	95.1143	1.87018	newly 5 weeks cumul	16 731.	9516.77	{1256., 19 465.}	101.77	0.792999	newly 59 weeks cumul	91 561.	10 003.1	{1458., 22 253.}	89.075	1.38772
